# A Simplified Calibration Procedure for DEM Simulations of Granular Material Flow

**DOI:** 10.3390/ma17194833

**Published:** 2024-09-30

**Authors:** Rashid Hajivand Dastgerdi, Agnieszka A. Malinowska

**Affiliations:** Faculty of Geo-Data Science, Geodesy, and Environmental Engineering, AGH University of Krakow, 30-059 Kraków, Poland; amalin@agh.edu.pl

**Keywords:** discrete element method, granular materials, micromechanical parameters, sand, repose angle test, cylinder test, ANSYS/LSDYNA

## Abstract

The discrete element method (DEM) has emerged as an essential computational tool in geotechnical engineering for the simulation of granular materials, offering significant advantages over traditional continuum-based methods such as the finite element method (FEM) and the finite difference method (FDM). The DEM’s ability to model particle-level interactions, including contact forces, rotations, and particle breakage, allows for a more precise understanding of granular media behavior under various loading conditions. However, accurate DEM simulations require meticulous calibration of input parameters, such as particle density, stiffness, and friction, to effectively replicate real-world behavior. This study proposes a simplified calibration procedure, intended to be conducted prior to any granular material flow DEM modeling, based on three fundamental physical tests: bulk density, surface friction, and angle of repose. The ability of these tests, conducted on dry quartz sand, to accurately determine DEM micromechanical parameters, was validated through numerical simulation of cylinder tests with varying height-to-radius ratios. The results demonstrated that this calibration approach effectively reduced computational complexity while maintaining high accuracy, with validation errors of 0% to 12%. This research underscores the efficacy of simplified DEM calibration methods in enhancing the predictive reliability of simulations, particularly for sand modeling in geotechnical applications.

## 1. Introduction

The discrete element method (DEM) is a vital computational tool for modeling granular materials in geotechnics, offering advantages over continuum-based methods by explicitly simulating particle-level interactions. DEM captures the discrete nature of granular media, including contact forces and particle rotations, providing insights into material behavior under various loading conditions. However, accurate DEM simulations require precise calibration of input parameters such as particle stiffness, friction, and damping. Determining these microscale parameters, such as contact properties, is challenging because direct experimental measurement is often infeasible. Calibration techniques using numerical tests have been developed to iteratively adjust parameters until the simulated bulk response matches experimental observations. 

The angle of repose test has been widely used to determine inter-particle sliding and rolling friction coefficients. Ucgul et al. [1] and Aikins et al. [2] applied this test to align simulated soil flowability and surface profiles with experimental data. Salazar et al. [3]; Xu, Liu, and Yang [4]; Benmebarek and Movahedi Rad [5]; Benmebarek et al. [6]; and Hajivand Dastgerdi et al. [7] used the direct shear test in their calibration processes to determine friction and stiffness parameters in their numerical simulations. Wilson and Sáez [8] employed cyclic torsional shear tests to refine friction coefficients, enhancing the precision of their DEM models. Jeong et al. [9] analyzed shear and particle crushing characteristics in a ring shear system using DEM, calibrating parameters through ring shear tests under varying conditions. Chung and Ooi [10] used confined compression and rod penetration tests to validate DEM simulations, adjusting inter-particle friction and particle stiffness to match experimental bulk compressibility data. For industrial applications, Ketterhagen and Wassgren [11] utilized a ring shear tester to calibrate DEM models, focusing on accurately representing bulk flow behavior. Johnstone [12] developed a calibration methodology for DEM models by using bulk physical tests, such as a rotating drum for the dynamic angle of repose and confined compression tests for bulk stiffness. This approach involved a parametric optimization procedure to fine-tune DEM parameters. This process aligned simulation results with experimental data to enhance model accuracy. Coetzee [13] and Coetzee and Els [14] employed compression testing to quantify the stiffness of DEM particles, followed by the determination of friction parameters through direct shear testing. These derived parameters were subsequently integrated into a blade–soil interaction model. Despite some divergence from experimental outcomes, their study suggested the potential of this methodology for advancing DEM simulations of granular materials. Jensen, Fraser, and Laird [15] introduced three efficient calibration models for improving the accuracy of DEM simulations of dry granular material flow. Their methodology incorporated a bulk density test, a surface friction test for particle–surface friction coefficients, and an angle of repose test for inter-particle friction coefficients. These sequential tests, executed using LS-DYNA software, facilitated a streamlined calibration process, optimizing both runtime and parameter accuracy. Aikins et al. [2] provided a comprehensive review of calibration techniques, emphasizing the reliability of angle of repose, direct shear, and triaxial compression tests in DEM simulations. Yan et al. [16] for agricultural applications, used the direct shear test and response surface methods in their DEM model calibration process. Zhang et al. [17] developed a GPU-accelerated DEM simulator validated through various tests, including angle of repose and cone penetration, to accurately simulate complex granular behavior. Liu et al. [18] calibrated DEM parameters for soda saline soil using angle of repose and direct shear tests, supported by advanced statistical methods. Wu et al. [19] focused on improving soil-traction performance predictions by calibrating DEM parameters through cylinder tests and shear stress–displacement curves. Chen and Martinez [20] applied DEM to model soil penetration influenced by root circumnutation, using triaxial compression tests for calibration. Coetzee [21] reviewed both bulk and direct measurement approaches for DEM calibration, underscoring the need for multiple tests to achieve precise parameterization. Coetzee and Scheffler [22] conducted a review of calibration methods for DEM parameters in bulk modeling of cohesive materials. They categorized various methodologies, including angle of repose tests, shear tests, and other flowability tests, highlighting the necessity of using multiple experiments to obtain a unique set of DEM parameters and avoid non-unique solutions.

In this study, a series of simple physical tests was conducted to determine key micromechanical parameters for discrete element method (DEM) modeling, focusing on friction coefficients and particle density. The parameters obtained from the calibration tests were subsequently validated using cylinder tests with varying height-to-radius ratios. The aim was to develop a straightforward procedure for accurately capturing the flow behavior of granular materials in DEM simulations.

## 2. Discrete Element Method in LS-DYNA

**Figure 1:**
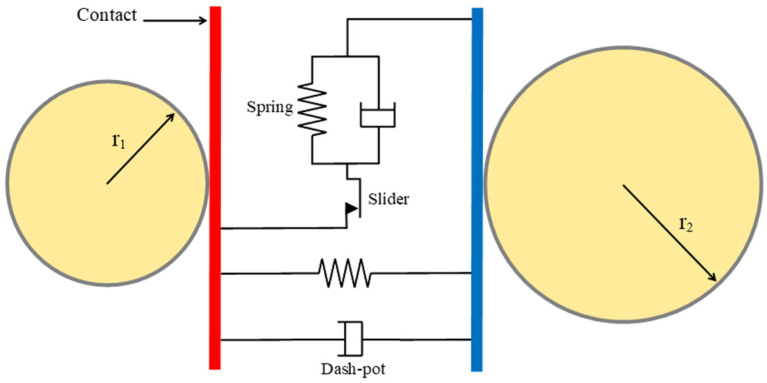
Particle–particle interaction.

The discrete element method (DEM), as implemented in LS-DYNA, is grounded in the framework initially proposed by Cundall and Strack [23]. Within this approach, granular materials are modeled as assemblies of rigid particles, with their interactions determining the bulk behavior of the material. The translational and rotational motions of the particles are computed at each time step based on Newton’s laws of motion [24]. The particle interactions are governed by contact mechanics using penalty-based contact algorithms, allowing the specification of normal and tangential stiffness, damping, and both static and rolling friction coefficients [25]. Figure 1 provides a schematic of the particle contact interaction. The detailed formulation of the contact model can be found in the literature [26,27,28].

## 3. Calibration Approach

Various calibration methods have been employed to determine the micromechanical parameters for DEM simulations. Among these, direct shear tests have frequently been utilized alongside compression tests or triaxial tests. These models, due to their extensive dimensions and the need to simulate particles with high stiffness, are computationally intensive, especially because the calibration process involves repetitive iterations. Additionally, considerable effort is required for their laboratory execution. However, for granular flow modeling, simpler tests are available that offer faster and more straightforward models. These tests do not necessitate the simulation of high-stiffness particles, thereby allowing the use of larger time steps in DEM simulations, significantly reducing computational time.

**Figure 2:**
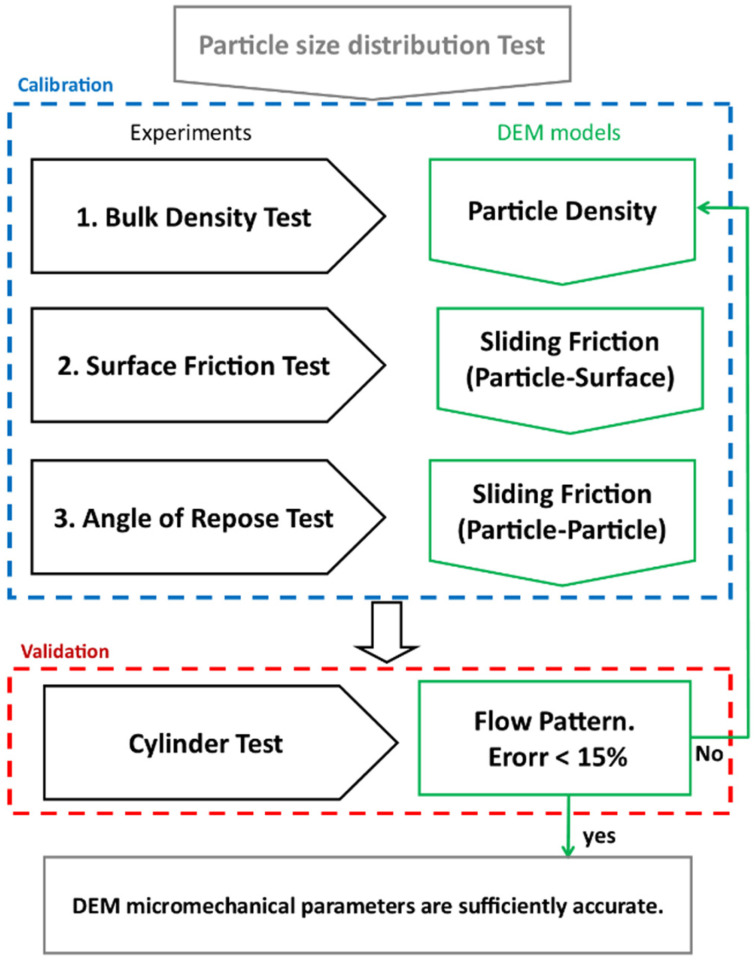
Flowchart of the calibration process.

In this study, the calibration of DEM parameters for modeling granular material flow was systematically conducted by integrating three fundamental physical tests with corresponding numerical simulations. These tests; bulk density, surface friction, and angle of repose, were originally recommended by Jensen et al. [15] as key assessments in their industrial DEM projects. In our research, the physical execution and numerical modeling of these tests were slightly refined to enhance the simplicity of both the lab test execution and the simulation. The validity of the calibrated parameters was evaluated through simulations of cylinder tests. Detailed descriptions and justifications for each test are provided in the subsequent sections, with the overall DEM calibration process depicted in Figure 2. The calibration procedure was structured so that each physical test primarily relied on the output of the preceding experiment, ensuring a logical and efficient progression. For example, the bulk density test, where the influence of friction parameters was minimal, was performed first to accurately determine particle density. Establishing particle density first provided a reliable foundation for calibrating the friction parameters in later stages, with each test building upon the validated data from the previous one. As non-spherical particles were utilized to represent the shape of sand grains, as outlined in the subsequent section, the rolling friction coefficient was inherently addressed by the grain geometry. Accordingly, in the simulations, the rolling friction coefficient (μr) was set to a low value of 0.1 for all tests. The only parameters requiring determination through the simulations were the inter-particle and particle-surface sliding friction coefficient (μ) and the particle density.

**Figure 3:**
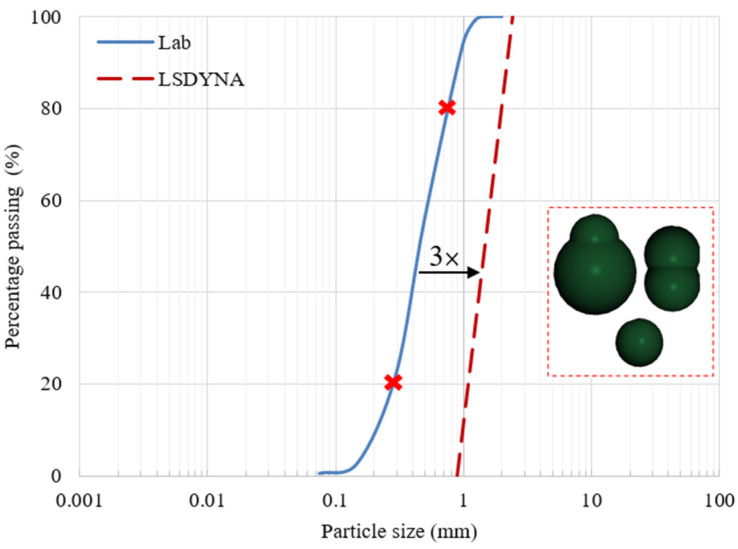
Particle size distribution curve from the laboratory test, along with the particle shapes and size distribution used in the simulations.

A dry quartz sand with a median grain size of 0.45 mm was used in this study. To reduce computational time in 3D discrete element method (DEM) models, an upscaling technique was applied to increase sand grain size. In granular flow simulations, identifying the smallest geometric dimension that particles interact with, such as the outlet diameter in funnels or hoppers, is crucial. Previous studies (Karajan et al., Jensen et al., and Feng et al.) recommended ensuring that at least five to eight grains can pass through the outlet simultaneously [15,26,29]. In this study, the 12 mm funnel outlet served as the limit for grain-size upscaling. The sand primarily consisted of grains retained between sieves with apertures ranging from 0.3 mm to 0.8 mm, corresponding to D20 and D80. Grains within this size range were selected for upscaling. To ensure that at least five grains passed simultaneously through the 12 mm funnel outlet in the angle of repose test simulation, a maximum grain size of 2.4 mm was calculated. Dividing this by the actual maximum grain size of 0.8 mm resulted in an upscaling factor of 3. Real sand is characterized by a variety of grain shapes, from semi-spherical to non-spherical. In the simulations, three different particle shapes, with dimensions ranging from 0.9 to 2.4 in their longest axis, as depicted in Figure 3, were utilized to more accurately reflect the grain geometry.

The main properties of the quartz sand used in the study are listed in Table 1.

### 3.1. Bulk Density Test

In granular material flow modeling, particle density is crucial compared with tests involving significant force and pressure, such as direct shear and triaxial tests. This is because, in flow modeling, the primary loads applied to the particles are due to gravity and the resulting weight of the grains. Therefore, having an accurate particle density is essential for precise simulations. Although particle (solid) density can be experimentally measured, in numerical simulations, an upscaling method is often employed to reduce computational time. Therefore, the particle density must be determined through simulations of the upscaled grains. This experiment was designed as a straightforward procedure for determining the particle density, using a principal-shaped container (cubic or cylindrical) with dimensions of approximately 5 cm to minimize computational time in numerical simulations. A cylindrical steel container, 41 mm in height and 61 mm in diameter, was filled with quartz sand, vibrated to achieve densification, and carefully leveled to ensure a flat surface. The mass of the sand within the container was then measured. The sand’s weight was recorded as 191 g.

**Figure 4:**
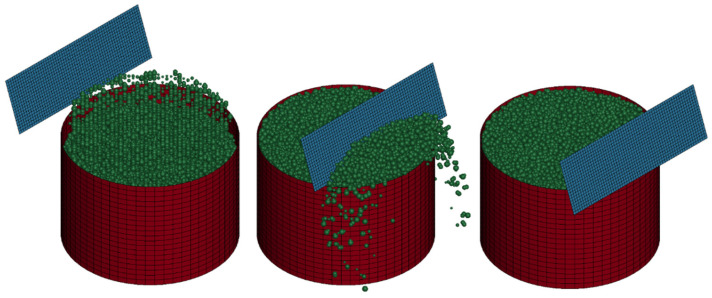
Bulk density model at various stages.

Following the physical experiment, and once the upscaling factor was selected as described in the previous section, the simulation was performed using ANSYS/LSDYNA software. An initial estimate of particle density was used, and the simulation was run to measure the simulated sand mass within the box using the discrete element method (DEM). There was a direct correlation between the input particle density and the resulting sand mass in the simulation. The DEM simulation of the bulk density test, similar to the physical experiment, comprised three distinct phases. First, the box was overfilled with sand and vibrated to achieve densification. Next, once the model reached equilibrium, the surface was leveled using a blade. Finally, after the leveling process was completed and equilibrium was maintained, the remaining sand mass was recorded. Figure 4 illustrates the bulk density model at different stages of the simulation.

In the simulation of the bulk density test, default friction parameters for sand were employed according to the literature. Additionally, to reduce the runtime of DEM models during the simulation of granular flow problems, it is recommended to use a stiffness value lower than the actual material stiffness, particularly for stiff materials like sand. For DEM modeling of sand under significant forces, such as those encountered in laboratory direct shear tests, the stiffness of sand particles is typically assumed to exceed 2 GPa. However, this high stiffness significantly increases the runtime by reducing the incremental time steps, which can considerably prolong the simulation process for granular flow tests due to their dynamic nature. In LS-DYNA, contact stiffness is determined by the parameters of the MAT_ELASTIC card, including Young’s modulus (E) and Poisson’s ratio (v). The elastic parameters must be selected so that increasing the particle stiffness does not alter the particle density. This approach allows for larger time steps, leading to faster calculations while preserving the accuracy of the models.

Based on the bulk density experiment and its corresponding DEM model, a particle density of approximately 2600 kg/m^3^ was determined by assuming a Young’s modulus of 1 GPa and a Poisson’s ratio of 0.22 for the sand particles. These parameters were be utilized in the following phases of the calibration process.

### 3.2. Surface Friction Test

This test aims to determine the particle-surface sliding friction coefficient. Each surface requires testing to obtain the corresponding friction parameter. In this study, smooth steel surfaces were used. Sand was poured onto the steel surface, allowed to settle, and then the surface was gradually tilted until the sand began to slide. In our test, the critical angle of 29 degrees, where motion initiated, was recorded and was applied in the DEM simulation.

**Figure 5:**
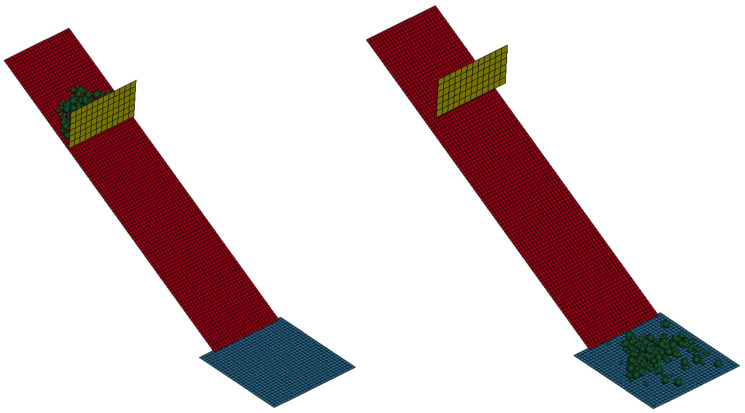
Stages of the DEM model for surface friction test.

In the DEM simulation using ANSYS/LSDYNA, a blade and plate setup were created, with sand poured onto the top section of the plate and the blade positioned to confine the sand. After equilibrium was reached, gravity vectors were adjusted in two directions to simulate a 29-degree inclination without physically rotating the plate, simplifying the model and reducing runtime. The model was then rebalanced under inclined conditions. Initial estimates for the particle-surface sliding friction coefficient, along with particle density and stiffness values from previous tests, were used. The blade was slowly raised, allowing the grains to move downward. If the grains remained stationary after removing the blade, it indicated that the assumed friction parameter was higher than the target value. The correct friction parameter was identified when a 0.05 increase caused the particles to remain stationary without sliding. The sliding friction coefficient was determined to be 0.55 through the surface friction test simulation. Figure 5 illustrates the simulation process.

### 3.3. Angle of Repose Test

**Figure 6:**
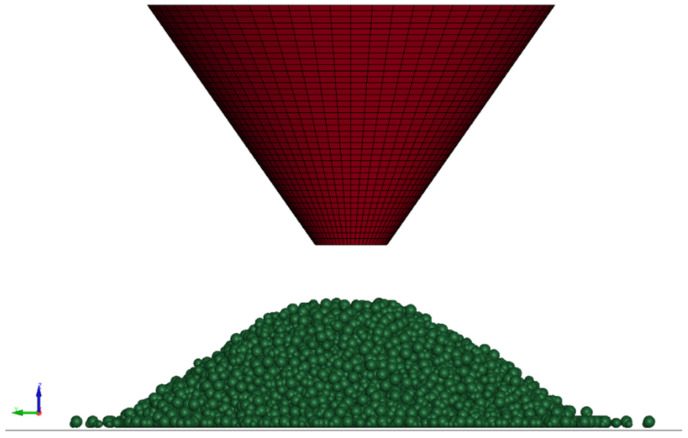
DEM model of the angle of repose test showing the final induced angle of the poured sand.

This test aimed to determine the inter-particle sliding friction coefficient. The setup involved pouring sand through a funnel onto a plate, with the resulting angle of repose measured after the sand settled. The average repose angle, recorded after three trials, was 34 degrees. The DEM simulation incorporated particle-surface sliding friction, particle density, and stiffness values from prior tests. Initial estimates of the sliding friction parameter were refined by comparing the simulated angle of repose with laboratory measurements. The simulation, which resulted in an angle of repose of approximately 34 degrees, identified the inter-particle sliding friction coefficient as 0.6, while the rolling friction coefficient was previously set at 0.1. Figure 6 illustrates the simulated test.

## 4. Validation of DEM Parameters

The DEM micromechanical parameters obtained from the previous tests were validated by simulating two cylinder tests and comparing the numerical results with experimental data.

### Cylinder Test

This test investigates the flow behavior of granular materials, such as sand, by observing the collapse of a vertical granular column within a hollow cylinder as the cylinder is gradually lifted [30,31,32,33,34]. This method is particularly effective for calibrating and validating DEM models, ensuring that the simulations accurately replicate the physical behavior of granular flows. The specifications for the cylinder tests are detailed in Table 2.

**Figure 7:**
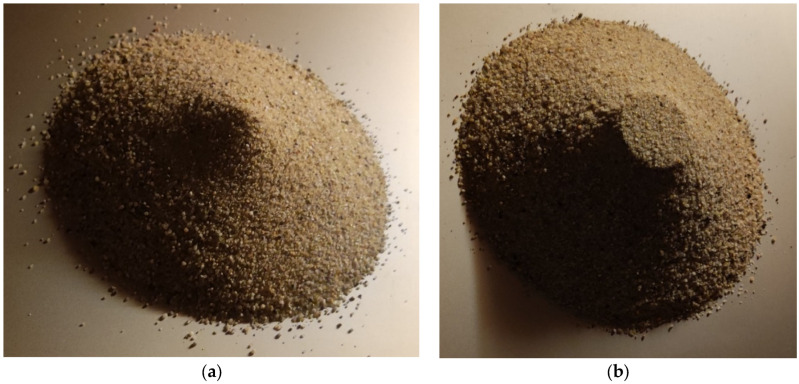
Sand repose after cylinder test for (**a**) a = 4.5 and (**b**) a = 0.625.

The flow pattern of the sand after performing the cylinder tests is shown in Figure 7.

**Figure 8:**
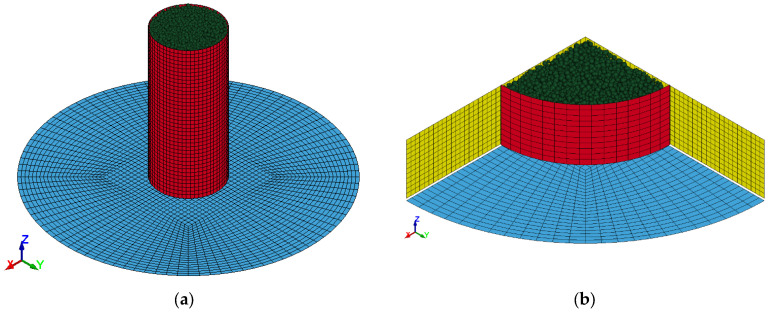
DEM numerical models built for both cylinder test scenarios. (**a**) a = 4.5 and (**b**) a = 0.625.

DEM numerical models were developed using micromechanical parameters derived from the sand calibration tests and the corresponding test geometries. Figure 8 presents the DEM models created for both cylinder tests using ANSYS/LSDYNA software. In case 2, to minimize computational time, only a quarter of the model was simulated, given the extended runtime caused by the higher number of particles and the symmetrical conditions.

## 5. Results and Discussion

The final calibrated micromechanical parameters for the sand under bulk flow conditions, determined through the calibration process, are presented in Table 3. Damping coefficients were applied in all models at their default values for sand modeling, as recommended in the literature [26].

**Figure 9:**
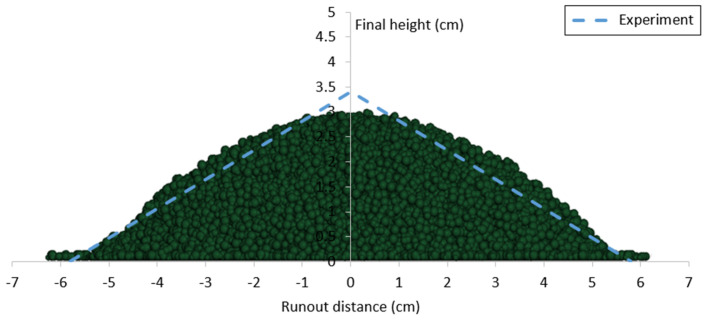
Comparison of repose shape and final deposit height between DEM simulation and experiment for case 1 (a = 4.5).

**Figure 10:**
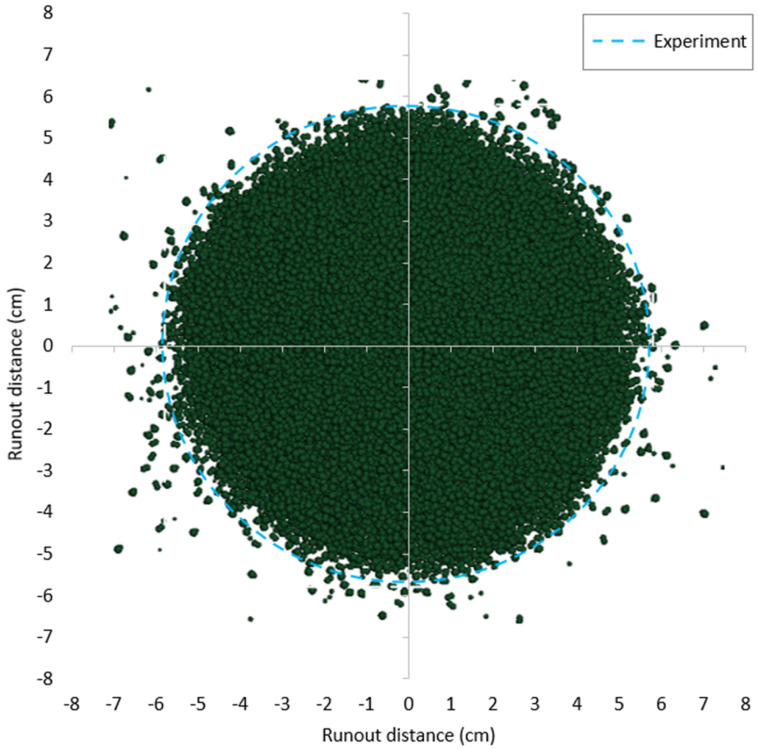
Comparison of final runout distance between DEM simulation and experiment for case 1 (a = 4.5).

**Figure 11:**
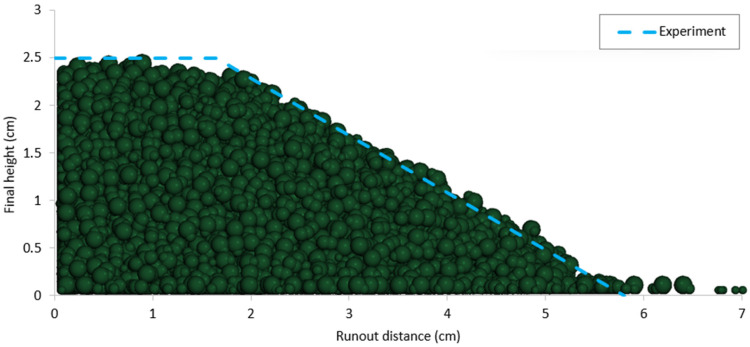
Comparison of repose shape and final deposit height between DEM simulation and experiment for case 2 (a = 0.625).

**Figure 12:**
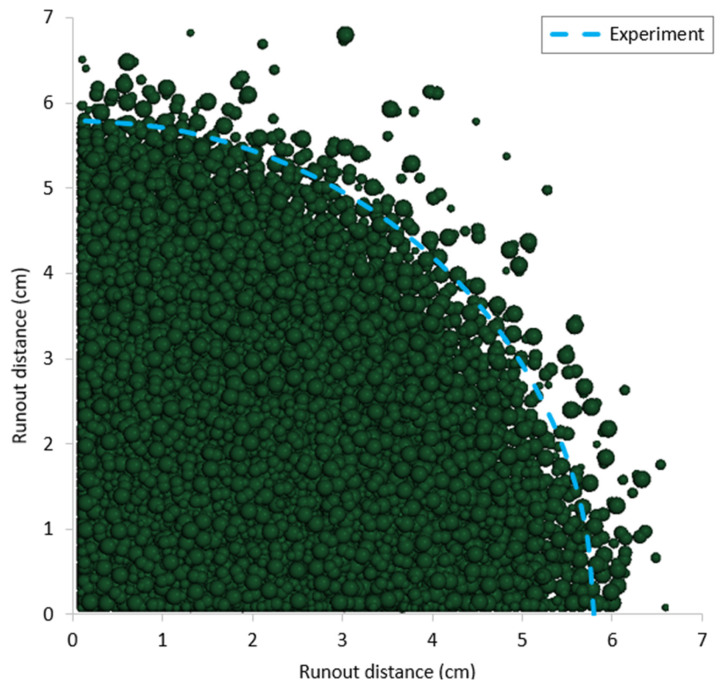
Comparison of final runout distance between DEM simulation and experiment for case 2 (a = 0.625).

Based on the calibrated DEM parameters, two cylinder tests were simulated using data from the experiments conducted on the same sand. The results, including comparisons of runout distance, final deposit height, and flow patterns between the experimental and DEM numerical outcomes, are presented in Figure 9, Figure 10, Figure 11 and Figure 12.

Table 4 demonstrates the differences between the conducted cylinder tests and their DEM simulations. As demonstrated in Table 4, the validation results exhibited a high level of accuracy, with an error range of 0% to 12%. These findings suggest that the three fundamental tests—bulk density test, surface friction test, and repose angle test—are effective for the calibration process of granular materials in granular flow modeling, especially for sand in geotechnical contexts.

Slight discrepancies observed between experimental and numerical simulation results can be attributed to particle upscaling and the shapes of particles used in DEM simulations. Studies reveal that in DEM models, finer and more angular particles enhance stability and interlocking, resulting in sharper and better-defined deposit edges [35,36,37,38,39].

## 6. Conclusions

This study demonstrates the effectiveness of utilizing basic physical tests—bulk density, surface friction, and angle of repose—to calibrate discrete element method (DEM) parameters for modeling granular material flow. DEM simulations of these tests were conducted to determine particle density and sliding friction between particles and surfaces. The calibrated parameters were subsequently applied to two cylinder tests, which served as validation controls to assess the accuracy of the parameters in the context of granular flow modeling.

The developed calibration procedure demonstrated sufficient accuracy, with validation tests showing error margins ranging from 0% to 12%. These results indicate that the proposed simplified calibration technique can significantly reduce computational time while maintaining the reliability of DEM simulations.

For future research, it is recommended to perform additional granular flow experiments, such as the drum mixer test, to further validate and refine the proposed calibration approach. Moreover, simulations should incorporate real-sized particles or employ lower upscaling factors, along with utilizing more complex and angular particle shapes, to enhance the assessment of the calibration method’s accuracy. 

## Figures and Tables

**Table 1:** Key properties of the quartz sand.

Property		Value
Bulk density [ϒ]	kg/m^3^	1591
Angle of internal friction [ɸ]	°	38
Cohesion [c]	kPa	7

**Table 2:** Specifications for the cylinder tests.

Case ID	h_0_	r_0_	a = h_0_/r_0_
	Height (cm)	Radius (cm)	Aspect Ratio
1	9	2	4.5
2	2.5	4	0.625

**Table 3:** Calibrated micromechanical parameters for DEM simulations in LS-DYNA.

	Symbol	Parameters	Value
Particle	Radii	Particle size [mm]	0.45–1.2
	RO	Density [kg/m^3^]	2600
	E	Young’s modulus [N/m]	1 × 10^9^
	ν	Poisson’s ratio	0.22
	NDAMP	Normal damping coefficient	0.7
	TDAMP	Tangential damping coefficient	0.7
	FRICS	Sliding friction coefficient	0.6
	FRICR	Rolling friction coefficient	0.1
	NORMK	User-defined normal spring constant	0.01 (default value) *
	SHEARK	User-defined shear spring constant	0 (default value) *
Particle surface	FricS	Sliding friction coefficient	0.55
	FricD	Rolling friction coefficient	0.1
	DAMP	Damping coefficient	0.7

* The software will calculate these parameters according to the stiffness parameters entered.

**Table 4:** Comparison of experimental and numerical results for cylinder tests.

Case ID	Runout Distance [r_ꝏ_]			Final Height [h_ꝏ_]		
	Experiment (cm)	DEM Model (cm)	Difference (%)	Experiment (cm)	DEM Model (cm)	Difference (%)
1	5.8	5.6	3.5	3.4	3	12
2	5.8	5.9	0	2.5	2.5	0

## Data Availability

The original contributions presented in the study are included in the article, further inquiries can be directed to the corresponding author.
